# Bioinformatics reveals TNFAIP6 as a candidate gene and suggests its potential crosstalk in the treatment of hemodialysis in chronic kidney disease

**DOI:** 10.1080/0886022X.2025.2528757

**Published:** 2025-07-13

**Authors:** Guoxin Zhang, Jieqiong Fu, Wenming Niu

**Affiliations:** Hemodialysis Room (Jianhua Clinic), Shijiazhuang, Hebei, China

**Keywords:** Hemodialysis, chronic kidney diseases, KRAS signal pathways, TGF-β signal pathway, hub gene

## Abstract

Hemodialysis (HD) is a life-sustaining treatment for chronic kidney disease (CKD) patients. This study aimed to identify candidate diagnostic biomarkers associated with HD-treated CKD. The public dataset was acquired from the Gene Expression Omnibus database. The differentially expressed genes (DEGs) showing opposite trends across the three groups were obtained as crosstalk genes, and their potential pathways were explored through KEGG and GSEA analyses. Next, hub genes were identified using LASSO regression, and their diagnostic potential was assessed *via* ROC analysis. Key immune cell populations were identified using ssGSEA. Blood samples from healthy controls (*n* = 10), CKD patients (*n* = 9), and HD-treated CKD patients (*n* = 7) were collected to validate hub gene expression. A total of 132 crosstalk genes were identified, with the TGF-β and KRAS signaling pathways potentially activated in the HD group. Two hub genes, SIK1 and TNFAIP6, exhibited AUC values exceeding 0.8 for diagnosing CKD and HD-treated CKD groups. Compared to the other groups, neutrophil abundance was significantly higher in CKD group and showed a strong correlation with the hub genes. External datasets and RT-qPCR validated a consistent expression trend of TNFAIP6. Therefore, TNFAIP6 may represent a potential candidate gene with biomarker relevance in CKD and HD-treated CKD. TNFAIP6 has been previously associated with the TGF-β pathway and neutrophil regulation, and its crosstalk mechanism in HD-treated CKD warrants further exploration.

## GRAPHICAL ABSTRACT

**Figure:**
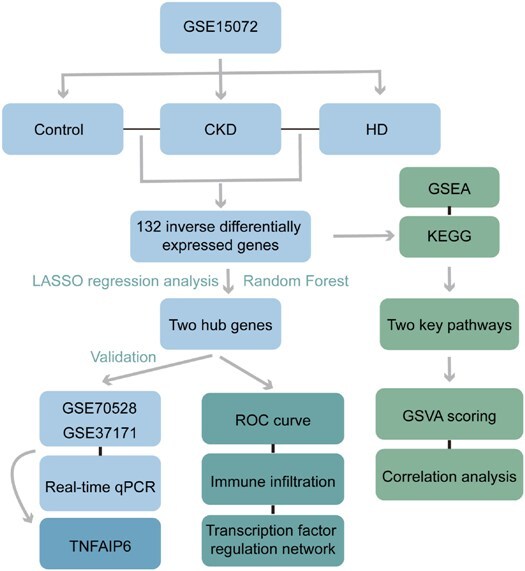

## 1. Introduction

Chronic kidney disease (CKD) is a progressive condition characterized by the loss of glomerular function, resulting in impaired kidney performance, of which end-stage renal disease (ESRD) is the last stage [1,2]. Various factors contribute to the development of CKD, such as hypertension, diabetes, obesity, female sex, and advanced age (>60 years) [1]. The global burden of CKD is estimated at 3–18% [3]. The estimated glomerular filtration rate (eGFR) and albumin-to-creatinine ratio (ACR) are commonly used screening measures for CKD diagnosis [4]. Renal fibrosis and inflammation are key pathological processes in the progression of CKD, contributing to the gradual deterioration of kidney function [5]. Research focusing on these mechanisms has contributed to the identification of potential therapeutic targets and diagnostic biomarkers [6]. For example, elevated levels of transforming growth factor-β1 (TGF-β1) have been proposed as an indicator of renal fibrosis in patients with progressive kidney disease and are associated with worse renal outcomes [7,8]. Additionally, the inflammation-related gene TNFAIP6 exhibits significantly altered expression in fibrotic kidney tissues and may serve as a diagnostic indicator of renal fibrosis progression [9].

In clinical practice, the main strategies to delay the progression of CKD focus on symptomatic treatments, including control of urinary protein, blood pressure, blood glucose, and lipids, as well as correction of complications. Renal replacement therapies, such as dialysis (hemodialysis (HD) and peritoneal dialysis) and renal transplantation, are also employed [10]. HD is a therapy utilizing extracorporeal circulation of the patient’s blood and is employed for both acute and chronic renal failure [11]. It is a rapid and effective treatment as it can correct disturbances in water, electrolyte, and acid-base balance and remove toxins from the body [12]. However, patients with HD treatment have a series of complications [13]. HD patients exhibit immune dysfunction, manifested as chronic inflammation responses, resulting in an elevated risk of infection, cardiovascular disease, and malignant tumors [14–16]. Understanding the molecular mechanisms underlying HD therapy in CKD patients is essential for identifying promising biomarkers and improving treatment outcomes.

In this study, transcriptomic data from healthy controls, CKD patients, and CKD patients treated with HD were obtained from a public database. Bioinformatics analyses were conducted to explore the underlying molecular mechanisms that showed different patterns among the three groups, including the hub genes, key pathways, and important immune cell populations. Through this analysis, we aimed to identify candidate genes that may reflect treatment-associated responses or HD-related improvements in CKD pathology, thereby providing potential biomarkers and improving disease management.

## 2. Materials and methods

### 2.1. Data collected and preprocessing

The GSE15072 dataset was included in our study after searching the keywords ‘chronic kidney disease’ and ‘Homo sapiens’ in the Gene Expression Omnibus (GEO, https://www.ncbi.nlm.nih.gov/geo/) database. The number of peripheral blood mononuclear cell (PBMC) samples in both datasets was not <3, and samples in both datasets included healthy controls, CKD, and HD groups. The GSE15072 dataset consisted of eight healthy controls, nine patients with stage II-III CKD, and 12 CKD patients treated with HD, provided by Granata et al. and obtained from platform GPL96. Patient selection for this dataset was based on strict inclusion and exclusion criteria, with detailed clinical information available in previous studies [17]. Furthermore, the GSE70528 dataset was obtained to validate gene expression, including 19 PBMC samples from eight controls (hypertension), seven stage III-IV CKD, and four HD patients (platform GPL570). Additionally, to define the impact of CKD stage on gene expression, we also included the GSE37171 dataset, which includes peripheral blood from 40 healthy controls, 17 end-stage renal failure patients, and 35 end-stage renal failure patients treated with HD (platform GPL570). Expression values at the probe level were converted to gene-level expression values. When multiple probes corresponded to the same gene, their average expression value was used to represent the gene-level expression. The expression matrix was log_2_-transformed for subsequent analyses.

### 2.2. Differential expression analysis

The GSE15072 dataset was divided into two groups: eight controls and nine CKD patients, as well as nine CKD patients and 12 HD samples. The CKD- and HD-related differentially expressed genes (DEGs) with *p*-value <0.05 and |log_2_ fold change (FC)| >1 were obtained from two groups using the ‘limma’ package. The results were visualized by volcano plots through the ‘ggplot2’ package. Then, the crosstalk genes between up- and down-regulated genes in both two groups were acquired.

### 2.3. Functional enrichment analysis

Kyoto Encyclopedias of Genes and Genomes (KEGG) enrichment analysis of crosstalk genes was performed through the DAVID database (https://david.ncifcrf.gov/) [18,19]. The terms with *p*-value <0.05 were selected and visualized.

### 2.4. Gene set enrichment analysis (GSEA)

GSEA was performed to explore the biological functions among healthy controls, CKD, and HD samples. According to the ‘h.all.v2022.1.Hs.symbols.gmt’ in the Molecular Signatures Database (MSigDB, https://www.gsea-msigdb.org/gsea/msigdb/), the gene sets with the false discovery rate (FDR) <0.25 and *p*-value <0.05 were selected [20]. Then, the enrichment results of gene sets were calculated in each sample using the ‘GSVA’ package (v1.30.3) of R. The differences in enrichment scores of different pathways were compared among control, CKD, and HD samples by the Wilcox test. The Pearson correlation analysis was performed among different pathways.

### 2.5. Identification of the hub genes

The machine learning algorithm was used to select hub genes. First, the random forest algorithm in the ‘randomForest’ package of R was utilized to rank crosstalk genes based on their average decline in accuracy between the two groups. Then, the top 25% of crosstalk genes were acquired and overlapped to obtain both CKD- and HD-related intersection genes. Next, the intersection genes were subjected to identify CKD- and HD-related key genes by the least absolute shrinkage and selection operator (LASSO) regression analysis using the ‘glmnet’ package of R. Finally, the CKD- and HD-related key genes were intersected to obtain both the CKD- and HD-related hub genes. The expression levels of hub genes were displayed using the box plot. The diagnostic values of hub genes were assessed using the receiver operating characteristic (ROC) curve created with ‘pROC’ in R.

### 2.6. Immune infiltration analysis

The immune cell landscapes among control, CKD, and HD samples were profiled through single-sample GSEA (ssGSEA) to quantify the relative abundance of each immune cell. The gene sets of immune cells were acquired from Charoentong’s study [21]. The immune infiltration levels among control, CKD, and HD samples were compared using the Wilcox test. Then, the association between hub genes and immune cells was explored by Pearson’s correlation analysis.

### 2.7. Transcription factor regulatory network

The transcription factors (TFs) regulating the hub genes were predicted using the Cistrome DB database (http://genemania.org). Transcription factors with a Regulatory Potential Score >0.5 were selected. The ‘TF-mRNA’ regulatory network was then constructed and visualized using Cytoscape.

### 2.8. RT-qPCR

Total RNA was extracted from 26 blood samples (10 healthy controls, 9 CKD patients, and 7 HD patients) using HiPure Liquid RNA/miRNA Kit (Magen, R4163-02). The clinical information of all samples is presented in Table S1. The cDNA was synthesized from mRNA transcription using FastQuant cDNA First Strand Synthesis Kit (Tiangen, KR116). The expression levels of SIK1 and TNFAIP6 were detected using RT-qPCR through the Gene-9660 real-time PCR system (BIOER, China) with SuperReal PreMix Plus (SYBR Green, Tiangen, FP205). GAPDH and ACTB were used as internal controls to normalize target gene expression levels. The PCR reaction system was programmed as follows: initial denaturation at 95 °C for 15 min, followed by 40 cycles of 95 °C for 10 s, 55 °C for 30 s, and 72 °C for 32s, and then a final extension at 95 °C for 15 s, 60 °C for 60s, and 95 °C for 15 s. The sequences of primers are displayed in Table S2. Primer efficiencies were calculated using a standard curve generated from serial dilutions of cDNA, and all primers showed efficiencies between 90% and 105%. Results were visualized using GraphPad Prism (v8.3.0, www.graphpad.com, USA), and statistical differences between the three groups were assessed using one-way ANOVA followed by Tukey’s *post-hoc* test.

### 2.9. Statistical analysis

All statistical analyses were conducted using R and GraphPad Prism (v8.3.0). Differences in gene expression and immune cell infiltration between groups were assessed using the Wilcox test. Correlation analyses were performed using Pearson’s correlation coefficient. To account for multiple comparisons, p values were adjusted using the Benjamini-Hochberg method (FDR). Unless otherwise indicated, p-values were treated as descriptive. Differences in RT-qPCR results among groups were evaluated using one-way analysis of variance (ANOVA) followed by Tukey’s *post hoc* test.

## 3. Results

### 3.1. Identification of DEGs among control, CKD, and HD samples

**Figure 1.**
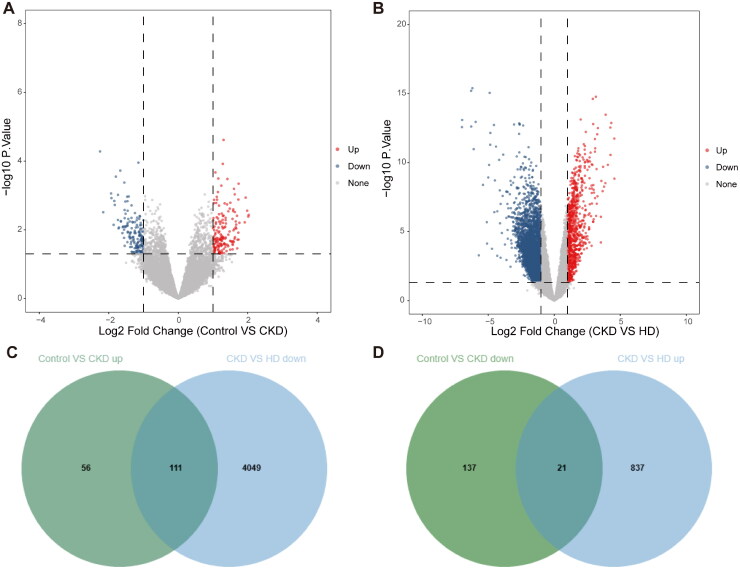
Identification of DEGs and crosstalk genes. (A,B) Volcano plots of DEGs in the training set in healthy controls *vs.* CKD patients (A) and CKD *vs.* HD-treated CKD patients (B). (C,D) Venn diagram of 111 (C) and 21 (D) crosstalk genes.

A total of 325 DEGs, including 167 up- and 158 down-regulated, were attained in patients with CKD compared with healthy controls (Figure 1(A)). Compared with CKD patients, a total of 5081 DEGs, including 858 up- and 4160 down-regulated, were acquired in CKD patients treated with HD (Figure 1(B)). Then, the 167 up-regulated DEGs were intersected with 4160 down-regulated DEGs, and 111 crosstalk genes were identified (Figure 1(C)). Similarly, the 158 down-regulated DEGs were intersected with 858 up-regulated DEGs to identify 21 crosstalk genes (Figure 1(D)). Finally, a total of 132 crosstalk genes were obtained.

### 3.2. TGF-β and KRAS may be key pathways in HD-treated CKD

**Figure 2.**
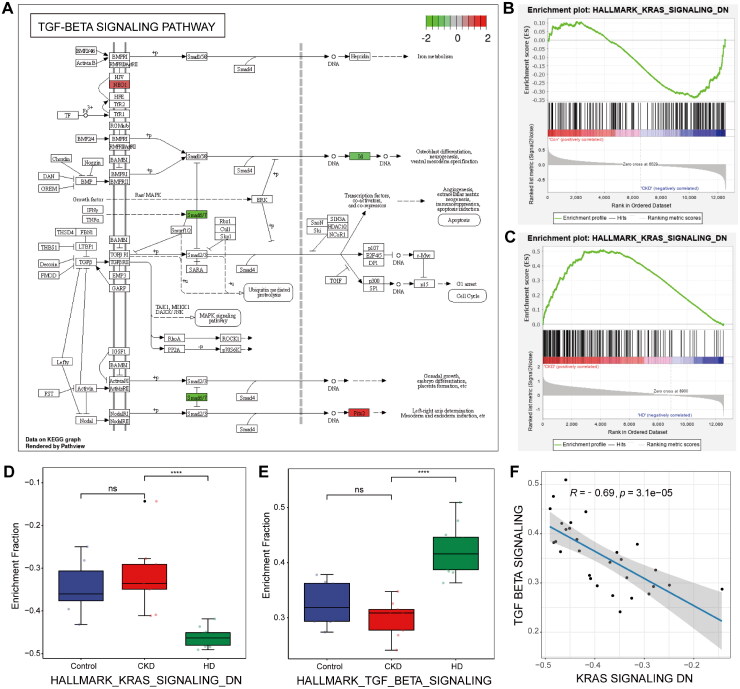
KEGG and GSEA analyses. (A) KEGG analysis of crosstalk genes showing the significant enrichment of TGF-β signaling pathway. The red represented the expression of genes that were increased in CKD group compared with healthy controls or HD group, while the green represented the expression of genes that were decreased. (B,C) The GSEA analysis showed enrichment score of the HALLMARK_KRAS_SIGNALING_DN in both the control *vs.* CKD groups (B) and CKD *vs.* HD groups (C). The green curve represents the enrichment score across the ranked gene list. Each vertical black line indicates the position of a gene from the gene set within the ordered dataset. The color bar in the middle reflects the correlation of gene expression with the phenotypes, with red representing positive correlations and blue indicating negative correlations. The bottom portion shows the ranking metric scores, where the zero crossing point indicates the position of peak enrichment. (D,E) The normalized enrichment score (NES) of KRAS-down (D) and TGF-β (E) signal pathways among control, CKD, and HD groups. Each point in the plot represents a sample’s NES. ****p < 0.0001; ns: no significant. (F) The correlation between KRAS and TGF-β signal pathways. Each dot represents a single sample, plotted based on the enrichment scores in all samples.

To further investigate the biological functions involved in 132 crosstalk genes, functional enrichment analysis was performed. KEGG enrichment analysis showed significant enrichment of the TGF-β signaling pathway (hsa04350, *p* = 0.023), involving the genes ID1, PITX2, NEO1, and SMAD7 (Figure 2(A)). The GSEA was utilized to explore the change in signal pathways among control, CKD, and HD samples, and the results revealed significant enrichment of the HALLMARK_KRAS_SIGNALING_DN (Figures 2(B,C)). The HALLMARK_KRAS_SIGNALING_DN gene set comprised 200 genes, while the HALLMARK_TGF_BETA_SIGNALING gene set included 52 genes. The enrichment scores of these gene sets were used to compare the differences in pathway activity across the three groups. The KRAS-down signal pathway was enhanced in the control *vs.* CKD groups and significantly reduced in the CKD *vs.* HD groups (Figure 2(D)). Furthermore, the TGF-β signal pathway demonstrated the opposite trend (Figure 2(E)). These results suggested that TGF-β and KRAS may be activated in CKD patients treated with HD. Additionally, Pearson’s analysis showed that there was a negative correlation between KRAS-down and TGF-β signal pathways of −0.69 (Figure 2(F)).

### 3.3. SIK1 and TNFAIP6 were the hub genes in both CKD and HD groups

**Figure 3.**
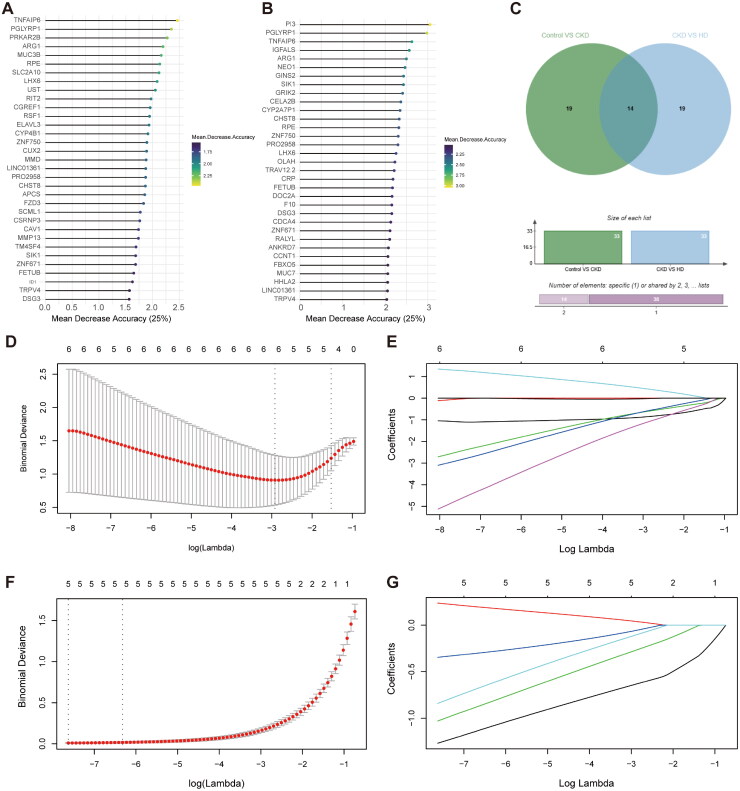
Identification of hub genes. (A,B) The mean decrease accuracy of 33 CKD-related (A) and 33 HD-related (B) crosstalk genes. (C) Venn plot of CKD- and HD-related intersection genes. (D,E) The 10-fold cross-validation to determine the optimal lambda in CKD (D) and HD (E) groups.

**Figure 4.**
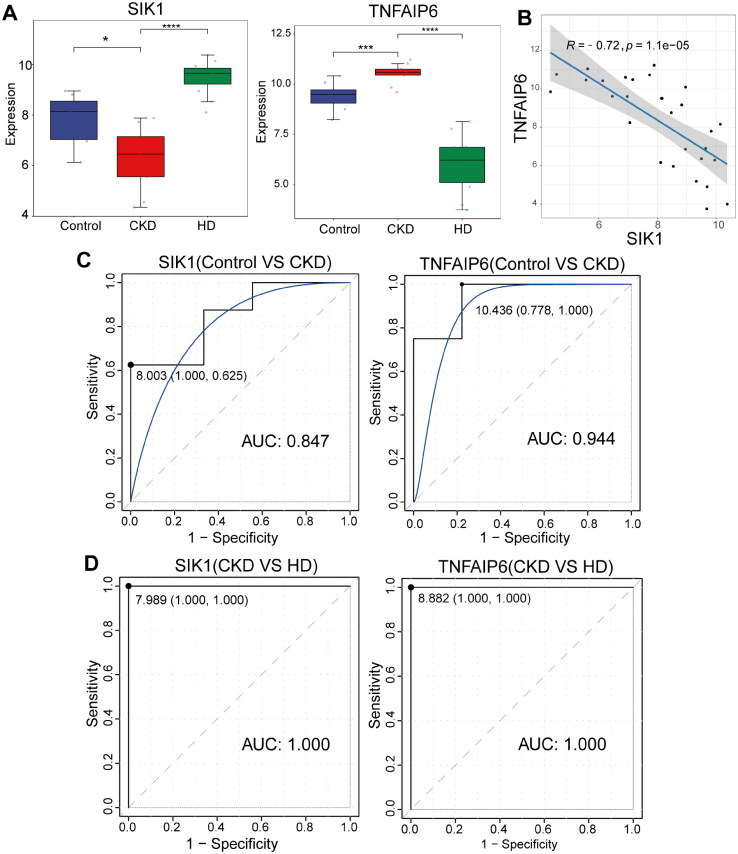
ROC analysis. (A) The expression levels of SIK1 and TNFAIP6 among control, CKD, and HD samples. *p < 0.05; ***p < 0.001; ****p < 0.0001. (B) The correlation between SIK1 and TNFAIP6. Each dot represents a single sample, plotted based on the gene expression levels in all samples. (C,D) The ROC analysis of SIK1 and TNFAIP6 in the control *vs.* CKD (C) and CKD *vs.* HD groups (D).

The importance of 33 (top 25%) CKD-related crosstalk genes and 33 (top 25%) HD-related crosstalk genes was illustrated in Figures 3(A,B). Then, a total of 14 CKD- and HD-related intersection genes were obtained (Figure 3(C)), including TNFAIP6, PGLYRP1, ARG1, RPE, LHX6, ZNF750, LINC01361, PRO2958, CHST8, SIK1, ZNF671, FETUB, TRPV4, and DSG3. The LASSO regression analysis showed that 5 CKD-related key genes (ARG1, LINC01361, PRO2958, SIK1, and TNFAIP6) and 5 HD-related key genes (PGLYRP1, SIK1, TNFAIP6, ZNF671, and ZNF750) were identified (Figures 3(D–G)). Then, SIK1 and TNFAIP6 were detected in both groups, determining them as the hub genes. The expression level of SIK1 was decreased in control *vs.* CKD groups, while in CKD *vs.* HD groups, SIK1 expression was increased (Figure 4(A)). In CKD patients, the expression level of TNFAIP6 was increased in both two groups (Figure 4(A)). Meanwhile, SIK1 and TNFAIP6 were negatively correlated with a coefficient of −0.72 (Figure 4(B)). ROC analysis showed that the AUCs of SIK1 and TNFAIP6 were 0.847 and 0.944, respectively, in control *vs.* CKD groups (Figure 4(C)). In CKD *vs.* HD groups, the AUCs of both SIK1 and TNFAIP6 were 1 (Figure 4(D)). These results indicated that SIK1 and TNFAIP6 could accurately diagnose CKD and HD-treated CKD patients.

### 3.4. Immune infiltration analysis

**Figure 5.**
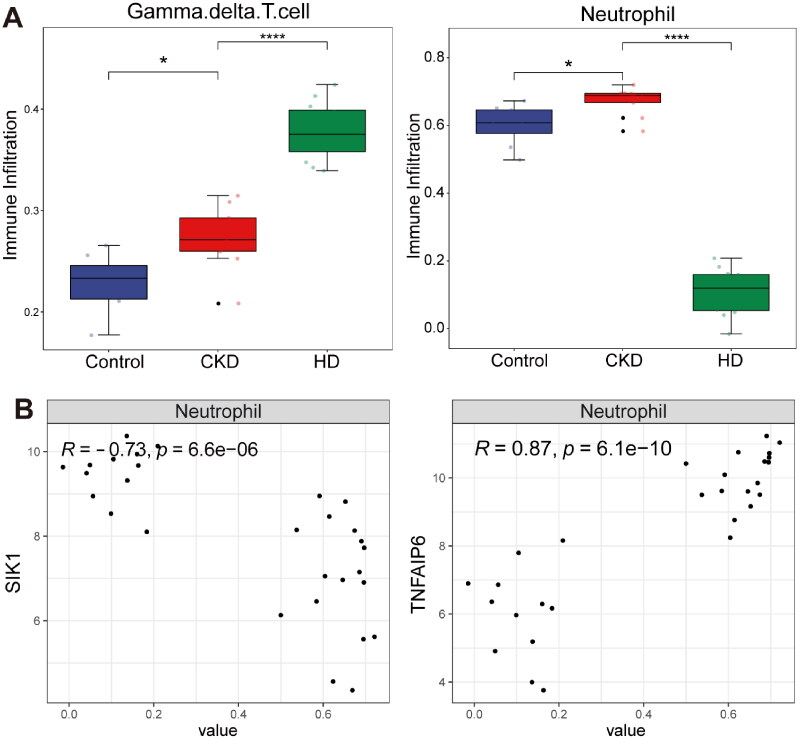
Immune infiltration analysis. (A) The abundances of gamma delta T cells (A) and neutrophils (B) among control, CKD, and HD groups. *p < 0.05; ^****^p < 0.0001. (B) The correlation between SIK1 and neutrophils, as well as between TNFAIP6 and neutrophils. Each dot represents a single sample, plotted based on the infiltration score of neutrophils (x-axis) and the expression level of the indicated gene (y-axis).

The results indicated that gamma delta T (γ-δT) cells and neutrophils in both control *vs.* CKD and CKD *vs.* HD groups had significant differences (Figure 5(A)). The immune infiltration level of gamma delta T cells was significantly increased in both two groups. The immune infiltration level of neutrophils was significantly increased in the CKD group, while it was significantly decreased in the HD group. Neutrophils exhibited an opposite trend in the control *vs.* CKD and CKD *vs.* HD groups, and we performed a correlation analysis between this immune cell and the hub genes. The results showed that neutrophils exhibited a negative correlation with SIK1 (*r* = −0.73) and a positive correlation with TNFAIP6 (*r* = 0.87, Figure 5(B)).

### 3.5. Transcription factor regulatory networks

**Figure 6.**
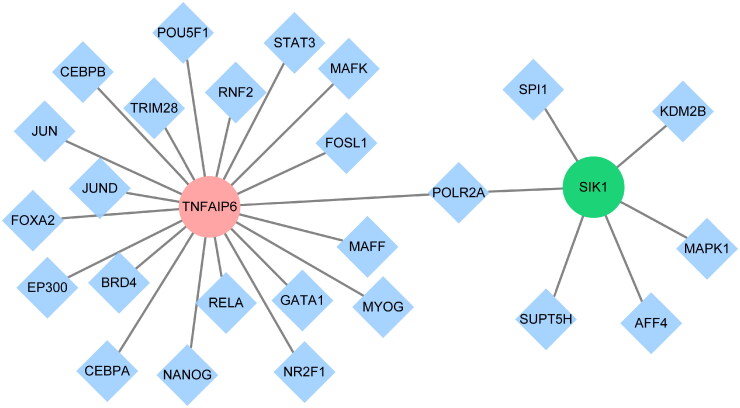
Construction of a transcription factor regulatory network. Diamonds represent transcription factors, and circles represent hub genes.

We constructed a ‘TF-mRNA’ regulatory network to explore potential upstream regulatory mechanisms (Figure 6). The analysis predicted that SIK1 is regulated by six TFs, while TNFAIP6 is regulated by 20 TFs. Among them, POLR2A was identified as a common regulatory factor for both hub genes.

### 3.6. Validation of the expression levels of SIK1 and TNFAIP6

**Figure 7.**
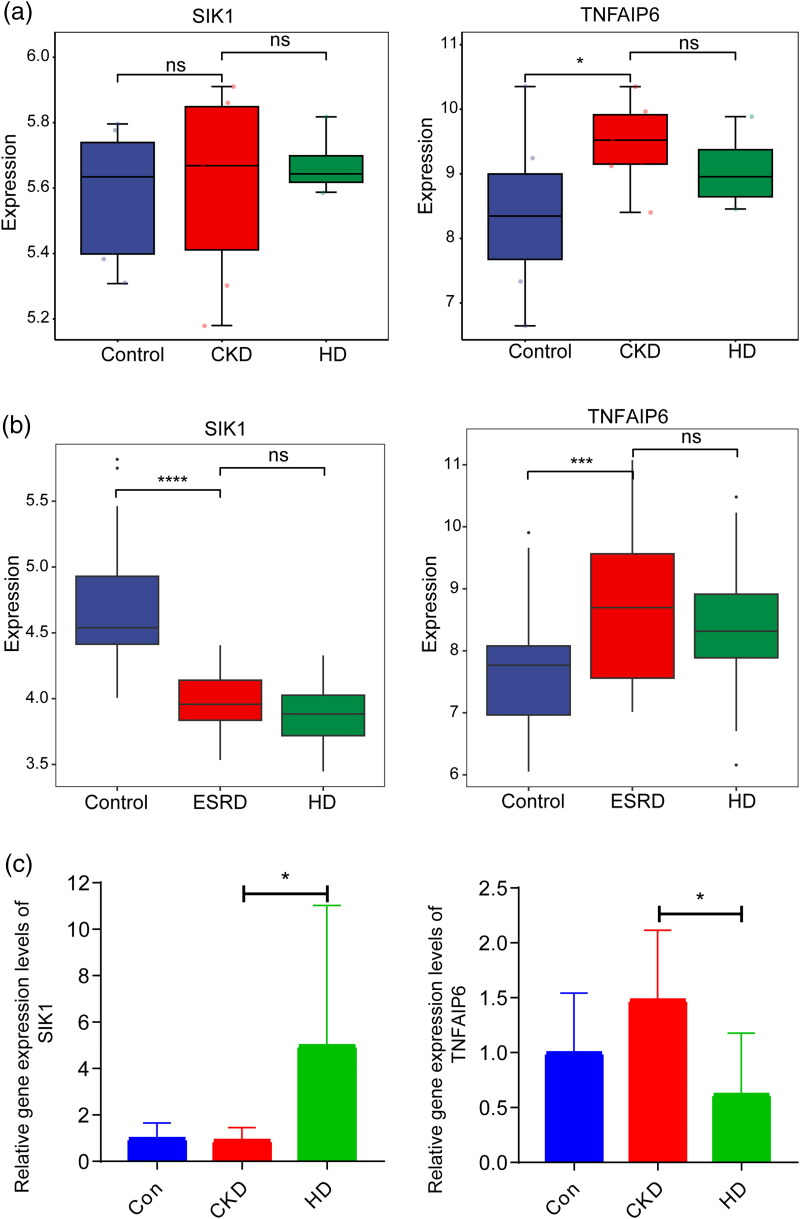
Expression validation of hub genes. (A,B) The expression levels of SIK1 and TNFAIP6 were validated in the GSE70528 (A) and GSE37171 (B) datasets. (C) The expression levels of SIK1 and TNFAIP6 were validated in healthy controls (*n* = 10), CKD patients (*n* = 9) and HD-treated CKD patients (*n* = 7) *p < 0.05; ***p < 0.001; ^****^p < 0.0001; ns: no significant.

We included datasets from different stages of CKD for external validation of SIK1 and TNFAIP6 expression, including GSE70528 (stage III-V CKD) and GSE37171 (stage V CKD, ESRD) (Figures 7(A,B)). The expression trend of TNFAIP6 in both datasets was consistent with that observed in the GSE15072 dataset. The expression levels of SIK1 and TNFAIP6 were assessed using RT-qPCR. The expression levels of SIK1 and TNFAIP6 were statistically significant among the CKD and HD groups (Figure 7). Although their expression was not statistically significant in the control and CKD groups, the observed expression trends were consistent with those in the GSE15072 dataset. Given the consistent expression pattern observed in multiple independent datasets, TNFAIP6 was considered a candidate gene as a biomarker for CKD and HD-treated CKD.

## 4. Discussion

HD is a widely used treatment for patients with kidney disease to remove accumulated metabolic waste products and excess fluids [22]. Through bioinformatics analysis, we identified 132 differentially expressed crosstalk genes among the control, CKD, and HD groups. Notably, two key signaling pathways, TGF-β and KRAS, along with a hub gene, TNFAIP6, and an immune cell population, neutrophils, were found to be potentially involved in HD-treated CKD.

Excessive extracellular matrix deposition leading to renal fibrosis is a major driver of CKD progression, with the TGF-β family playing a critical role in this process [23]. Moreover, this pathway regulates the function of various renal cell types and is actively involved in inflammatory responses, reflecting its pleiotropic role in CKD pathogenesis [24,25]. In patients undergoing HD, arteriovenous fistula (AVF) is a commonly used vascular access. Inhibition of TGF-β signaling has been shown to reduce endothelial-to-mesenchymal transition (EMT) and improve AVF patency in CKD mice [26]. Therefore, targeting the TGF-β pathway may enhance the therapeutic efficacy of HD in CKD patients. Our study found a significant negative correlation between the KRAS-down pathway and TGF-β. KRAS, a proto-oncogene GTPase belonging to the RAS superfamily of small GTPases, is inactivated through GDP binding under normal physiological conditions [27,28]. KRAS and TGF-β signal pathways may engage in crosstalk, collaboratively initiating the expression of EMT and fibrosis-related factors [29]. It has been shown that KRAS expression is elevated in CKD models, and silencing KRAS reduces TGF-βinduced fibrosis [30,31]. Therefore, we speculate that a synergistic mechanism between the KRAS and TGF-β pathways may contribute to the progression of renal fibrosis in CKD patients. Targeting these pathways may enhance the therapeutic efficacy of HD in CKD. Nevertheless, further validation is needed to confirm the underlying mechanisms of this potential interaction.

We initially identified two hub genes. Following external and PCR validation, the expression pattern of TNFAIP6 remained consistently aligned across datasets, suggesting that TNFAIP6 may serve as a key candidate gene associated with CKD and HD-treated CKD. TNFAIP6, encoding the tumor necrosis factor-α-stimulated gene-6 (TSG-6), mediates renal tubular inflammation and fibrosis through paracrine effects [32]. Blocking TNFAIP6 can inhibit EMT in renal epithelial cells [33]. Although no direct molecular interactions between TNFAIP6 and the KRAS pathway have been reported, they may be indirectly associated through mechanisms, such as the disease microenvironment, inflammatory signaling, or the TGF-β fibrosis program. An AUC ≥0.8 is commonly used as a heuristic threshold indicating good diagnostic performance [34]. In our study, TNFAIP6 demonstrated good diagnostic accuracy in distinguishing CKD and HD-treated CKD, with favorable sensitivity and specificity. Overall, TNFAIP6 may be a candidate gene with the potential to serve as a biomarker, offering utility in monitoring disease progression and informing treatment decisions. However, further validation in larger, prospective cohorts is warranted to confirm its clinical applicability.

Immune cell dysregulation plays a central role in renal fibrosis and CKD progression [35]. Our study revealed significant differential expression of neutrophils among healthy controls, CKD patients, and HD-treated CKD patients. Consistent with our findings, renal biopsies from CKD patients show significant neutrophil infiltration [36]. Neutrophils contribute to renal fibrosis through inflammatory cytokine release, neutrophil extracellular trap (NET) formation, and extracellular matrix deposition, ultimately impairing renal function [35,37]. Reducing neutrophil infiltration has been shown to alleviate renal injury [38]. However, it is important to note that HD itself, rather than kidney function decline, may contribute to neutrophil dysfunction [39], with evidence suggesting impaired chemotaxis in HD-treated CKD patients [40]. These findings emphasize the need for closer attention to neutrophil alterations in HD-treated patients. Our data demonstrate a significant correlation between TNFAIP6 and neutrophils, suggesting its potential regulatory roles in immune responses in CKD and HD-treated CKD patients. The association between TNFAIP6 and neutrophils has been elucidated in various diseases. TNFAIP6 knockout mice exhibit delayed wound healing and increased neutrophil accumulation [41]. In skin diseases, TNFAIP6 has been shown to suppress neutrophil recruitment by reducing STAT1 phosphorylation levels in keratinocytes [42]. Additionally, TNFAIP6 may play a key role in renal inflammation by interacting with CXCL8 to inhibit neutrophil transendothelial migration [43].

In the present study, TNFAIP6 was identified as a candidate gene that may serve as a biomarker for diagnosing CKD and HD-treated CKD. Future studies should validate TNFAIP6 expression in larger, stage-balanced cohorts to facilitate clinical translation and evaluate its association with treatment response and long-term prognosis. Prospective studies incorporating serial TNFAIP6 measurements before and after HD may help determine whether its expression dynamically reflects disease activity or therapeutic benefit. Integrating TNFAIP6 expression data with clinical parameters, such as age, estimated glomerular filtration rate (eGFR), inflammatory markers, and dialysis adequacy, may improve diagnostic accuracy and support individualized treatment strategies in HD-managed CKD [44]. Ultimately, functional studies are warranted to elucidate the mechanistic role of TNFAIP6 in CKD pathology, and these hypotheses require further exploration and validation in future clinical investigations.

Nevertheless, there were some limitations in our study. First, all available RNA-seq data were collected from the public database, and the sample size was limited due to difficulties in acquiring clinical samples. Furthermore, due to limitations in the clinical characteristics available in the public cohort, adjustment for these confounders was not conducted. The potential of TNFAIP6 as a biomarker requires validation in larger, stage-balanced cohorts with more comprehensive clinical data. Second, the associations between KRAS and TGF-β signal pathways in CKD patients treated with HD needed to be explored further. Third, hub genes were validated by RT-qPCR, and the biological functions and their relationship with signaling pathways need to be validated. Fourth, candidate genes were selected based on opposite expression trends, with the aim of better identifying potential biomarkers associated with CKD and HD treatment. However, this approach may not capture the full biological complexity of pathway crosstalk. Fifth, the selection criteria for DEGs and hub genes were designed to retain biologically significant signals while minimizing noise from non-informative variation in small datasets. Nevertheless, this strategy may overlook important molecular players involved in the disease process.

## 5. Future directions

Future research is warranted to further elucidate the interplay between genes, signaling pathways, and immune cells in the context of CKD and HD, which may contribute to a more comprehensive understanding of disease mechanisms and guide therapeutic strategies. To validate the biological role of TNFAIP6 and its potential interaction with the TGF-β and KRAS pathways, *in vitro* studies using kidney epithelial or fibroblast cells should be performed to investigate the effects of TNFAIP6 modulation on fibrosis-related signaling. Although establishing chronic HD models in animals remains technically challenging [45,46], combining CKD models (e.g., 5/6 nephrectomy) with AVF surgery may offer a partial simulation of HD-related physiological alterations [26]. Additionally, generating TNFAIP6-knockout models, if feasible, could help clarify its mechanistic involvement in renal fibrosis and assess its potential relevance in CKD progression and treatment response. These proposed studies will be essential to support its translation from a candidate gene to a clinically relevant biomarker. Accordingly, TNFAIP6 expression may aid in the detection of CKD progression, stratification of patients for HD initiation, and dynamic monitoring of treatment efficacy and long-term prognosis.

## 6. Conclusion

In summary, we identified TNFAIP6 as a potential candidate gene with diagnostic relevance for CKD and HD-treated CKD. However, this study is exploratory, and further validation is required to confirm the broader clinical applicability of TNFAIP6. Additionally, our findings suggest potential involvement of the TGF-β and KRAS pathways, as well as neutrophils, in HD-treated CKD.

## Supplementary Material

Table S2.docx

Table S1.docx

Graphical abstract.tif

## Data Availability

The data in this study are available from the corresponding author upon reasonable request.
