# Supplementary material for: Bioinformatics reveals TNFAIP6 as a candidate gene and suggests its potential crosstalk in the treatment of hemodialysis in chronic kidney disease
Source: Ren Fail. 2025 Jul 13;47(1):2528757. doi: 10.1080/0886022X.2025.2528757 (PMC12261513; doi:10.1080/0886022X.2025.2528757)
Supplement: Table S2.docx [file IRNF_A_2528757_SM0154.docx]

**Table S2: Primer sequences**

| Name | Species | Forward primer | Reverse primer |
| --- | --- | --- | --- |
| GAPDH | Human | GGAGCGAGATCCCTCCAAAAT | GGCTGTTGTCATACTTCTCATGG |
| ACTB | Human | CATGTACGTTGCTATCCAGGC | CTCCTTAATGTCACGCACGAT |
| TNFAIP6 | Human | GTCTGTGCTGCTGGATGGAT | TAAAGACGCCACCACACTCC |
| SIK1 | Human | GCTTCTGAACCATCCACACAT | GTGCCCGTTGGAAGTCAAATA |
