# Supplementary material for: Bioinformatics reveals TNFAIP6 as a candidate gene and suggests its potential crosstalk in the treatment of hemodialysis in chronic kidney disease
Source: Ren Fail. 2025 Jul 13;47(1):2528757. doi: 10.1080/0886022X.2025.2528757 (PMC12261513; doi:10.1080/0886022X.2025.2528757)
Supplement: Table S1.docx [file IRNF_A_2528757_SM0153.docx]

**Table S1: The clinical information of all samples**

| Group | Number | Age | Gender | BMI (Kg/m^2^) | Hypertensive (mmHg) | eGFR | Duration of dialysis (years) | Primary Disease | Stages of CKD |
| --- | --- | --- | --- | --- | --- | --- | --- | --- | --- |
| 1 | 1 | 49 | Female | 25.4 | 110/80 | 102.4 | 0 |  |  |
| 1 | 2 | 52 | Male | 28.7 | 120/74 | 111 | 0 |  |  |
| 1 | 3 | 60 | Male | 24.2 | 104/75 | 104.2 | 0 |  |  |
| 1 | 4 | 64 | Female | 29.5 | 125/76 | 108.4 | 0 |  |  |
| 1 | 5 | 54 | Female | 23.6 | 114/70 | 118.6 | 0 |  |  |
| 1 | 6 | 55 | Male | 28.3 | 108/62 | 112.8 | 0 |  |  |
| 1 | 7 | 68 | Female | 25.4 | 110/65 | 104.8 | 0 |  |  |
| 1 | 8 | 59 | Female | 26.5 | 120/70 | 107.5 | 0 |  |  |
| 1 | 9 | 65 | Male | 26.7 | 125/74 | 103.6 | 0 |  |  |
| 1 | 10 | 50 | Female | 27.5 | 112/57 | 104.3 | 0 |  |  |
| 2 | 11 | 50 | Male | 27.8 | 120/65 | 52.3 | 0 | Hypertensive | G3 |
| 2 | 12 | 65 | Female | 28.7 | 120/70 | 54.8 | 0 | Hypertensive | G3 |
| 2 | 13 | 50 | Female | 24.5 | 110/75 | 48.7 | 0 | Hypertensive | G2 |
| 2 | 14 | 61 | Male | 23.8 | 114/70 | 70.4 | 0 | Renal arteriosclerosis | G2 |
| 2 | 15 | 58 | Male | 28.5 | 107/60 | 60.6 | 0 | Hypertensive | G2 |
| 2 | 16 | 66 | Male | 23.4 | 117/66 | 60.8 | 0 | Hypertensive | G2 |
| 2 | 17 | 58 | Female | 25.7 | 124/72 | 43.7 | 0 | Chronic nephritis | G3 |
| 2 | 18 | 42 | Male | 26.8 | 120/60 | 59.1 | 0 | Chronic nephritis | G3 |
| 2 | 19 | 57 | Female | 28.4 | 115/55 | 55.4 | 0 | Renal arteriosclerosis | G3 |
| 3 | 20 | 72 | Male | 24.3 | 145/69 | 14.5 | 2 | Hypertensive | G5 |
| 3 | 21 | 65 | Male | 26.2 | 150/65 | 5.4 | 1.5 | Hypertensive | G5 |
| 3 | 22 | 56 | Male | 23.9 | 130/78 | 18.3 | 7 | Chronic nephritis | G5 |
| 3 | 23 | 62 | Female | 24.3 | 120/85 | 14.1 | 5 | Chronic nephritis | G5 |
| 3 | 24 | 63 | Female | 24.2 | 135/65 | 7.5 | 5 | Renal arteriosclerosis | G5 |
| 3 | 25 | 53 | Male | 25.7 | 135/65 | 6.8 | 3 | Hypertensive | G5 |
| 3 | 26 | 56 | Female | 25.4 | 130/70 | 9.7 | 2 | Hypertensive | G5 |

1: Healthy controls, 2: Chronic Kidney disease (CKD) group, 3: Hemodialysis group, BMI: Body Mass Index, eGFR: Estimated Glomerular Filtration Rate, G: Glomerular Filtration Rate.
