# Supplementary figures and images for: Bioinformatics reveals TNFAIP6 as a candidate gene and suggests its potential crosstalk in the treatment of hemodialysis in chronic kidney disease
Source: Ren Fail. 2025 Jul 13;47(1):2528757. doi: 10.1080/0886022X.2025.2528757 (PMC12261513; doi:10.1080/0886022X.2025.2528757)

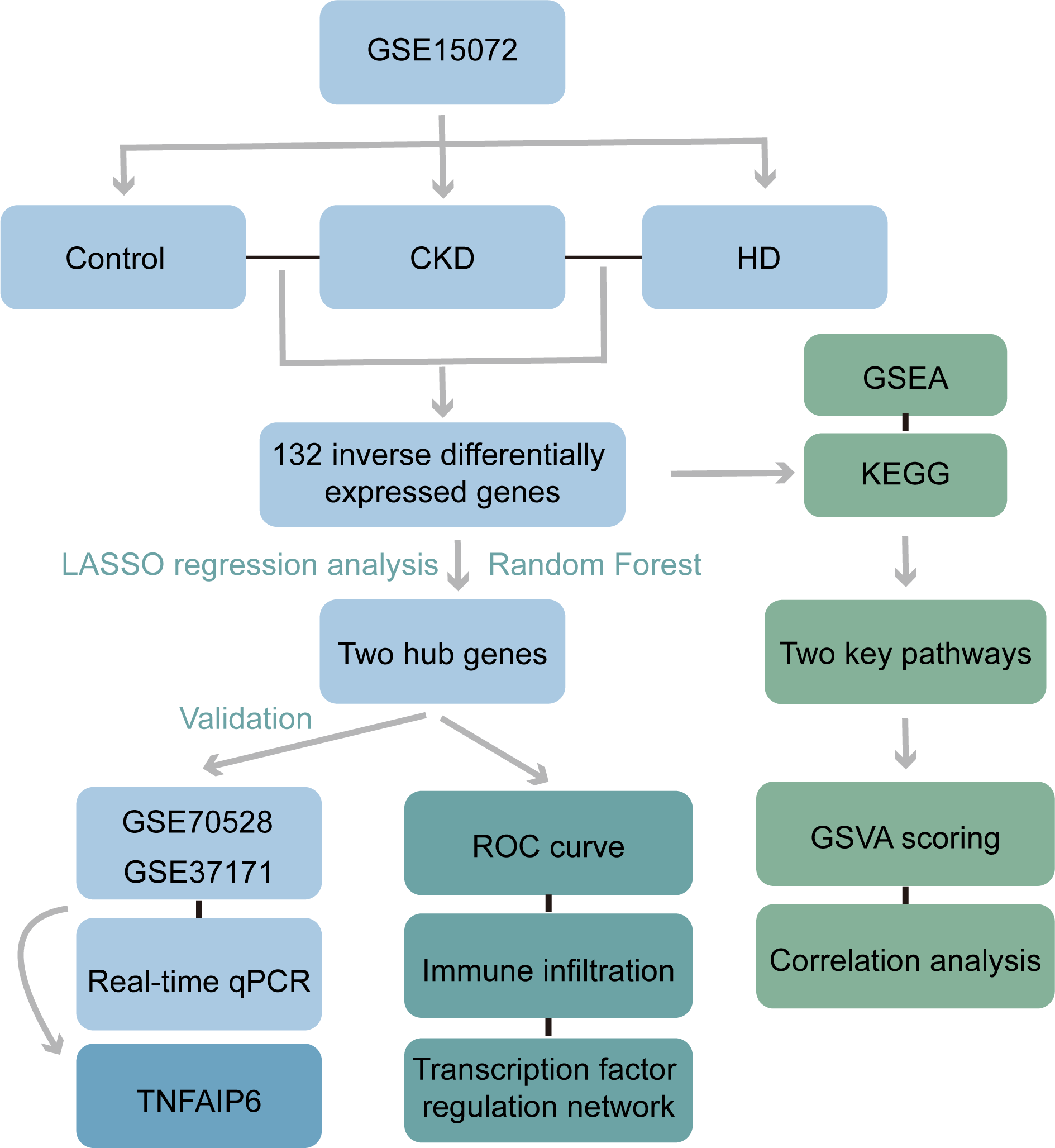

Supplement: Graphical abstract.tif [file IRNF_A_2528757_SM0152.tif]
